# Nuclear and plastid haplotypes suggest rapid diploid and polyploid speciation in the N Hemisphere ***Achillea millefolium ***complex (Asteraceae)

**DOI:** 10.1186/1471-2148-12-2

**Published:** 2012-01-03

**Authors:** Yan-Ping Guo, Shuai-Zhen Wang, Claus Vogl, Friedrich Ehrendorfer

**Affiliations:** 1 Ministry of Education Key Laboratory for Biodiversity Science and Ecological Engineering, and College of Life Sciences, Beijing Normal University, Beijing 100875, China; 2 Institute of Animal Breeding and Genetics, University of Veterinary Medicine, Vienna, A-1210 Vienna, Austria; 3 Faculty Centre of Biodiversity, Faculty of Life Sciences, University of Vienna, A-1030 Vienna, Rennweg 14, Austria

## Abstract

**Background:**

Species complexes or aggregates consist of a set of closely related species often of different ploidy levels, whose relationships are difficult to reconstruct. The N Hemisphere *Achillea millefolium *aggregate exhibits complex morphological and genetic variation and a broad ecological amplitude. To understand its evolutionary history, we study sequence variation at two nuclear genes and three plastid loci across the natural distribution of this species complex and compare the patterns of such variations to the species tree inferred earlier from AFLP data.

**Results:**

Among the diploid species of *A. millefolium *agg., gene trees of the two nuclear loci, ncp*GS *and *SBP*, and the combined plastid fragments are incongruent with each other and with the AFLP tree likely due to incomplete lineage sorting or secondary introgression. In spite of the large distributional range, no isolation by distance is found. Furthermore, there is evidence for intragenic recombination in the ncp*GS *gene. An analysis using a probabilistic model for population demographic history indicates large ancestral effective population sizes and short intervals between speciation events. Such a scenario explains the incongruence of the gene trees and species tree we observe. The relationships are particularly complex in the polyploid members of *A. millefolium *agg.

**Conclusions:**

The present study indicates that the diploid members of *A. millefolium *agg. share a large part of their molecular genetic variation. The findings of little lineage sorting and lack of isolation by distance is likely due to short intervals between speciation events and close proximity of ancestral populations. While previous AFLP data provide species trees congruent with earlier morphological classification and phylogeographic considerations, the present sequence data are not suited to recover the relationships of diploid species in *A. millefolium *agg. For the polyploid taxa many hybrid links and introgression from the diploids are suggested.

## Background

Species complexes or aggregates consist of a set of closely related species often of different ploidy levels, whose relationships are difficult to reconstruct. Such species complexes are common in angiosperms [1-5]. Rapid genetic, phenotypic and ecological differentiation on one hand, and hybridization/polyploidy on the other, play important roles in their evolutionary bursts [6-9]. The temperate N Hemisphere common yarrow taxa form such a complex, i.e., the *Achillea millefolium *aggregate. Centered in SE Europe and SW to C Asia, its diploid species are limited to Eurasia, whereas the polyploids have spread throughout the N Hemisphere [10,11]. In N America, the 4x and 6x cytotypes form a complex of ecological races adapted to many different niches with marked genotypic diversification [6,12,13]. By cultivation in experimental gardens, Clausen *et al*. [6] documented local adaptation of *A. millefolium *populations to environments along an altitudinal transect in California from sea level to alpine regions. This has become a classic example of rapid adaptive divergence of plant populations [12-18].

Our earlier AFLP data have characterized the *Achillea millefolium *complex as a clade [10]. The inferred species relationships of the diploid members conformed to a binary bifurcating tree and generally agreed with traditional species delimitations and taxonomic arrangements. The polyploid members appeared to be polytomic and polyphyletic although geographic patterns can be recognized [10,11]. Available data show that frequent exchange of genetic materials have been involved in the origins of many polyploidy taxa. Meanwhile, it seems also to have occurred during the divergence of the diploid species [10,11,19]. So far, we are still uncertain about the demographic history of *A. millefolium *agg. and the progenitors of many polyploid taxa.

Due to their dominant nature, AFLP markers are difficult to use for inferring genetic parameters of populations, particularly ancestral population sizes, split times, and migration rates. In principle, this can be accomplished better with DNA sequences either from organelles or from the nuclear genome [20,21]. Yet, we still meet challenges in practice: Plastid DNA variation is often too low to infer relevant gene trees with confidence. In addition, despite the lower effective population size of plastids, plastid gene trees may still not reflect the species tree due to incomplete lineage sorting. With nuclear genes, frequent birth and death of gene copies, less lineage sorting due to higher effective population size, secondary introgression among split species as well as intragenic recombination tend to hamper interpretation of the patterns of polymorphism [22-25].

Here, we survey DNA sequence variation sampled at two nuclear loci and three plastid fragments from populations across diploid, tetraploid, and hexaploid species throughout the natural distribution range of *A. millefolium *agg. and from three diploid congeneric species outside the aggregate. On the diploid level, we infer the demographic history among species and populations using the newly generated DNA sequence data in comparison to the relationships inferred from the previous AFLP data. To this end, we also apply a probabilistic model (IMa2) [26,27] to three widespread diploid species to shed light on the key parameters, i.e., ancestral effective population sizes, time of speciation, and rates of gene flow. For the polyploid populations, we try to untangle their polytomic and polyphyletic relationships which are probably complicated by gene flow on the same and between different ploidy levels using the co-dominant single-gene haplotype data.

## Methods

### Plant sampling

We sampled thirty populations of seven diploid, seven tetraploid and four hexaploid taxa or cytotypes of the *Achillea millefolium *aggregate throughout the temperate N Hemisphere (Table 1). On average, two to three individuals from each population were analyzed. Broadly the same individuals were sequenced for the two nuclear loci and three cpDNA fragments; minor exceptions were due to repeated failures in sequencing a certain locus from a certain individual (see Table 1). Three diploid species, taxonomically outside the *A. millefolium *aggregate but included in previous AFLP analyses [10], were also sampled for this study. They are the W-Eurasian *A. nobilis*-2x, the C-Mediterranean *A. ligustica*-2x and the East Asian *A. acuminata*-2x (Table 1). In addition, sequences of two cpDNA regions, *trnH-psbA *and *trnC-ycf6*, of 43 North American populations available from the NCBI data base (GenBank accessions EU128982-EU129456) [12] are incorporated into our plastid haplotype network analysis.

**Table 1:** Taxa, populations and DNA loci sampled

Taxa	Taxon abbrevi-ations	Pop. code	Ploidy	Geographic locality of populations	Collectors and dates	Nuclear genes: number of indiv./clones analyzed		cpDNA: number of indiv. analyzed
*Achillea millefolium agg*.						ncp*GS*	*SBP*	
*A. asiatica *Serg. s.lat. (= *A. sergievskiana *Shaulo & Shmakov)	asi-2x	NM	2x *^f^*	China: Daqing Mt., 41°04'52" N, 112°35'56" E; 2010 m	GR, 2006.08.25	3/8	3/9	4
		ARX	2x *^f^*	China: Arxan Mt., 47°17'39" N, 120°27'11" E; 1130 m	YG, 2007.10.08	3/14	3/14	3
		SHB	2x *^f^*	China: Hebei, 42°26' N, 117°15' E; 1500 m	YG, 2007.07.27	-	-	2
		AL1	2x *^c^*	Russia: Altai, 51°02'52" N, 85°36'47" E; 1100 m	MS, 2002.07.30	3/9	3/11	3
*A. asiatica *Serg. s.str.	asi-4x	AL9563	4x *^c^*	Russia: Altai, 49°32'66" N, 88°13'35" E; 2350 m	AT, 2003.08.02	3/13	1/5	3
		AL3	4x *^c^*	Russia: S Siberian lowland near Novosibirsk; 220 m	MS, 2002.08.16	3/13	3/15	2
		UT	4x *^c^*	Uzbekistan: Tashkent, Tschimgan Mt.	HG, 2002.11	-	3/15	3
*A. asplenifolia *Vent.	asp	BZ	2x *^c^*	Austria: Burgenland, Zitzmannsdorfer Wiesen	Tod, 2001.01.10	3/9	2/8	2
		NS2	2x *^c^*	Austria: Burgenland, Rust, near lake "Neusiedler See"	FE, JS, YG, 2003.05.27	3/9	3/15	3
		Ta	2x *^f^*	Czech Republic: South Moravia, Terezin	FE & LE, 2002.07.12	3/11	2/10	2
*A*. *borealis *Bong. s.lat. (*A. lanulosa *Nutt. var. *alpicola *Rydb.)	bor-alp	US2	4x *^i^*	USA: Washington, Mt. Rainier National Park; 1350-2070 m	PS & AT, 2002.08.18	2/12	2/10	2
*A. borealis *Bong. s.lat. (*A. lanulosa *Nutt. var.?)	bor-lan	US5	4x *^i^*	USA: Connecticut, Hopeville State Park	JE, 2004.07.17	2/12	2/10	2
*A. borealis *Bong. s.lat. (*A. lanulosa *Nutt. var. *lanulosa*)	bor-lan	US6	4x *^i^*	USA: Utah, ascent from Snowbird Alta to Lake Secret	KT, 2004.07.31	3/15	3/18	3
*A. ceretanica *Sennen s.str.	cer-2x	10240	2x *^c^*	France: E Pyrenees	JS, 2001	2/10	2/9	2
*A. ceretanica *Sennen s.lat.	cer-4x	10222	4x *^c^*	France: Massif Central	JS, 2001	2/10	2/9	2
*A. cuspidata *Wall.	cus	ID	2x *^i^*	Kashmir: 34°25.80' N, 75°44.80' E; 3200 m	LK, 2004.09.14	1/4	1/5	1
*A. distans *Waldst. & Kit. ex Willd.	dis	DIKF	6x *^i^*	Austria, Kaltenleutgeben, Flösslberge	JS, s.n.	2/16	2/13	2
*A. inundata *Kondr.	inu	K13	4x *^i^*	Ukraine: Kiev, S of Desna mouth into Dnjepr; 100 m	FE & YG, 2003.07.28	2/9	2/9	2
*A. latiloba *Ledeb. ex Nordm.	lat	Geo	2x *^i^*	Georgia: Adjara, 41°29'55″ N, 42°31'46″ E; 2006 m	DK, 2004.07.18	3/12	1/4	3
*A. millefolium *L. s.lat. (*A*. *apiculata *N.I.Orlova)	mil-api	Ra-c	6x *^i^*	Russia: Karelia, Louhski region, (a) Kandalaksha Natural Reserve; (b) Kiv bay, near Medvezhij peninsula; (c) near cape Ivanov Navolok	OA, 2003.08	5/32	5/37	5
*A. millefolium *L. s.lat. (*A*. s*udetica *Opiz)	mil-sud	STms	6x *^i^*	Austria: Salzburg, Hohe Tauern; ca. 2300 m	PS, 2002.08.31	2/10	2/15	2

*A. roseoalba *Ehrend.	ros-2x/ros-4x	Si3	2x+4x*^c^*	Slovenia: Ljubljana	FE, 2002.07.31	2/8	1/5	2
		Si6	2x+4x*^c^*	Slovenia: Ljubljana, Podpec	FE, 2002.7.31	2/8	1/5	2
		V	2x+4x*^c^*	Italy: Udine, Kanalta, MalborghettoValbruna	JS, 2002.07	3/15	2/10	3
*A. schmakovii *Kupr.	sch	AL5	6x*^c^*	Russia: Altai, 51°02'52'' N, 85°36'47'' E; 1700 m	MS, 2002.07.30	2/9	2/15	2
*A. setacea *Waldst. & Kit.	set	GR	2x *^i^*	Greece: Thessaloniki, drain from lake Limni Koronia	FE, 2001	1/3	1/5	1
		K4	2x *^c^*	Ukraine: Kiev, Bald Mt., Lisa Gora	FE & YG 2003.07.22	3/7	2/10	2
		NS1	2x *^c^*	Austria: Burgenland, E of St. Margarethen; ca 200 m	FE, JS, YG, 2003.05.27	2/8	2/7	2
		SeAA	2x *^c^*	Turkey: Anatolia, Aksaray	FE, 2002.03.26	3/10	2/10	2
*A. styriaca *J. Saukel & J. Danihelka, ined.	sty	StE	4x *^i^*	Austria: Styria, Einach, Wald	JS, s.n.	1/5	1/4	1
Species outside *A. millefolium *agg.								
*A. acuminata *(Ledeb.) Schultz Bip.	acu	CB1	2x *^f^*	China: Jilin, Changbai Mt., Hancong Valley, 680-620 m	YG & GR, 2002.07.24	3/8	3/8	
		ARX2	2x *^c^*	China: Inner Mongolia, Arxan, N 47°17'39.5", E 120°27'09.9"; 865 m	YG, 2007.10.08	3/12	2/11	
		TB5	2x *^c^*	China: Shanxi, Taibai Mt., N 34°01'17", E 107°18'21"; 1700 m.	YG, 2006.09.09	3/9	3/7	
*A. ligustica *Vis.ex Nym.	lig	SN	2x *^i^*	Italy: Sicily, Nebrodi Mts.	FE, 2001.09.21	1/3	1/5	1
*A. nobilis *L.	nob	ZN	2x *^i^*	Czech Rep.: Znoimo	LE & FE, 2002.07.13	3/24	1/5	

*^c ^*ploidy level checked by chromosome counting; ^*f *^ploidy level checked by flowcytometry; *^i ^*ploidy level inferred from FE's previous studies or from literature.

Names of collectors: AT = A. Tribsch; DK = D. Kharazishvili; FE = F. Ehrendorfer; GR = G.-Y. Rao; HG = H. Greger; JE = J. Ehrendorfer; JS = J. Saukel; KT = K. Tremetsberger; LE = L. Ehrendorfer-Schratt; LK = L. Klimes; MS = M. Staudinger; OA = O. Alexandrova; PS = P. Schönswetter; YG = Y.-P. Guo

To check ploidy levels of the populations studied, two methods, either chromosome counting or DNA ploidy level determination, were applied using young flower buds or fresh or silica gel-dried leaves, respectively. Young flower buds were collected in the field and fixed in Carnoy's fluid (ethanol:acetic acid = 3:1). To count the chromosome number, fixed flower buds were stained and squashed in 4% acetocarmine and observed under the microscope. DNA ploidy levels were investigated with propidium iodide flow cytometry [28,29] from the prepared leaves. Information for ploidy levels obtained with the above two methods are marked in Table 1 by *c *and *f*, respectively, while those inferred from previous studies [12 and Ehrendorfer *et al*., unpubl. Data] are marked by *i*.

Voucher specimens are deposited in the herbaria of the Institute of Botany (WU) and the Department of Pharmacognosy (HBPh), both at the University of Vienna, Austria, and of the College of Life Sciences, Beijing Normal University (BNU).

### Data sampling

Total genomic DNA was extracted from ca. 0.02 g silica gel desiccated leaf materials following the 2 × CTAB protocol [30] with slight modifications: Before the normal extraction process, sorbitol washing buffer was used to remove polysaccharides in the leaf materials (add 800 μL sorbitol buffer to the ground leaf powder → incubate the sample in ice for 10 min → centrifuge at 10,000 g for 10 min at 4°C and then follow the established 2 × CTAB protocol).

Two nuclear genes were sampled and partially sequenced for this study. They are the chloroplast-expressed Glutamine Synthase gene (ncp*GS*) and the Sedoheptulose-Bisphosphatase (*SBP*) gene. The ncp*GS *gene has been used in many plant phylogenetic studies and shown to be single-copy in all angiosperm species so far studied [31] and especially in *Achillea *[19]. We sequenced part of its coding and noncoding regions from exon 7 through to exon 11. The *SBP *gene has been studied in representative taxa of the family Asteraceae [32]. It was shown to be single-copy by our preliminary analyses in several diploid species of *Achillea*. Readers are referred to Ma *et al*. [19] for primers used for amplifying the ncp*GS *locus and to Chapman *et al*. [32] for the *SBP *locus (its exon 5 through to 7, i.e. the locus B12 in Chapman *et al*. 2007).

Three noncoding chloroplast DNA regions, *trnH-psbA*, *trnC-ycf6 *(including partial *ycf6-psbM*) and *rpL16 *were sequenced. PCR reactions were conducted with universal primer pairs [33].

The amplification was carried out in a volume of 20 μL with final concentration of 1 × PCR buffer, 0.05 U ex*Taq *(TaKaRa, Shiga, Japan) or HiFi (TransTaq DNA polymerase High Fidelity, TransGen Biotech), 200 μM of each dNTP, 1% DMSO, 0.5 μM of each primer, and with 1 μL template DNA and ddH_2_O added to the final volume. The amplification was conducted on a Peltier thermocycler (Bio-RAD) initiated with 5 min of pre-denaturing at 94°C followed by 30 cycles of 1 min at 94°C, 30s at 48-55°C, and 1.5 min at 72°C. A final extension was then taken at 72°C for 15 min followed by a hold at 4°C. The PCR products were electrophoresed on and excised from the 1.0% agarose gel in TAE buffer. They were then purified using a DNA Purification kit (TianGen Biotech or TransGen Biotech, Beijing, China). The purified PCR products were either used for direct sequencing (for the cpDNA fragments) or ligated into a pGEM-T Vector (for nuclear genes) with a Promega Kit (Promega Corporation, Madison, USA). For sequencing the nuclear genes, about five to eight positive clones from each diploid and ten to fifteen from each polyploid individual were randomly selected for sequencing. The plasmid was extracted with an Axyprep Kit (Axygene Biotechnology, Hangzhou, China). Cycle Sequencing was conducted using ABI PRISM^® ^BigDye™ Terminator. The same primers used for amplification (for cpDNA fragment) or the vector primers T7/Sp6 (for nuclear genes) were applied here. The sequenced products were run on an ABI PRISM™ 3700 DNA Sequencer (PE Applied Biosystems).

### Data analyses

Sequences were assembled with the ContigExpress program (Informax Inc. 2000, North Bethesda, MD), aligned with ClustalX 1.81, and then manually improved with BioEdit version 7.0.1. To prevent possible sequencing errors, single mutations in the nuclear gene data sets likely generated by the cloning sequencing method were excluded from the analyses. Furthermore, unique sequences in the nuclear gene data matrix, which do not fall into any majority-rule consensus sequence group [19,34] or show inconstant branch positions in trees based on different subsets of data, i.e., with partial characters or randomly selected sequences, during the initial analyses were eliminated to avoid influence of PCR-mediated recombination [19,35,36]. The final numbers of individuals/clones analyzed at each locus for each population are listed in Table 1. All the sequences analyzed were submitted to the NCBI GenBank under accession numbers HQ601971-HQ602593 (the nuclear ncp*GS *and *SBP *genes) and HQ450864-HQ451071 (the plastid loci).

The allelic data sets of the two nuclear genes, ncp*GS *and *SBP*, were analyzed separately, whereas sequences of three cpDNA fragments were combined as one locus.

Gaps in the nuclear data sets were treated as missing data, whereas each indel position (no matter how many nucleotide sites it contained) of the plastid data set was coded as a binary character (0/1 = A/C) using the program *GapCoder *[37].

As *A. millefolium *agg. consists of species with short evolutionary history [10,11], Neighbor Joining (NJ), Maximum Parsimony (MP) and Median-Joining network were applied to the present data. For the nuclear sequences, Neighbour Joining and Parsimony analyses were performed with MEGA 5.05 and PAUP* 4.0b10a, respectively. All nucleotide substitutions were equally weighted. Gaps were treated as missing data. We first analyzed data of the diploid species to show diversification of the gene lineages, and then of all the taxa to investigate relationships among the polyploids and diploids within *A. millefolium *agg. The NJ analysis was conducted with Kimura's 2-parameter distances [38] and bootstrapped with 1000 replicates. For the MP method, heuristic searches were performed using 1000 random taxon addition replicates with ACCTRAN optimization and TBR branch swapping. Up to 10 trees with scores larger than 10 were saved per replicate. The stability of internal nodes of the MP tree was assessed by bootstrapping with 1000 replicates (MulTrees option in effect, TBR branch swapping and simple sequence addition).

Median-Joining network analysis implemented in Network ver. 4.5.1.6 available at http://www.fluxus-engineering.com/sharenet.htm[39] was applied to the cpDNA data set. All variable sites were equally weighted and the homoplasy level parameter (ε) was set to zero given that variation rates of the closely related species is low, especially in their plastid DNA.

To understand the population demography at the time of speciation of the diploid species of *A. millefolium *agg., we applied a probabilistic model, the Isolation with Migration Model for multiple populations implemented in IMa2 [27], to three widespread and closely related species *A. asplenifolia*-2x and *A. setacea*-2x and *A. asiatica*-2x. These species are here regarded as three diverged populations which share nuclear sequence variation. Shared alleles could reflect ancestral polymorphism or gene flow after separation of the populations or species. Assuming neutrality, retention of ancestral polymorphisms is likely if speciation is fast relative to drift, which is inverse in intensity to the effective population size. As a rule of thumb, species are well separated with little ancestral polymorphism and thus almost complete lineage sorting, if the time of separation is at least as long as four times the effective population size [40]. Secondary genetic exchange between the diverging species can also lead to shared alleles observed [41]. The multipopulation model IMa2 allows both ancestral polymorphism and gene flow subsequent to divergence. It assumes a known history of the sampled populations, which can be represented by a rooted bifurcating tree. In earlier analysis using AFLP data [10], we inferred the rooted species tree as: ((*A. asiatica*, *A. asplenifolia*), *A. setacea)*. We note that Ima2 provides posterior distributions of parameters, such that the confidence in the inference of each parameter can be obtained from observing the spread of the posterior distribution. The IM model also assumes neutral genetic variation, freely recombining unlinked loci and no intragenic recombination or gene conversion [42]. Sequences of the two nuclear loci, the ncp*GS *and the *SBP *genes, and of three plastid fragments were used for this analysis. The polymorphic sites of the sequenced nuclear and plastid loci are mostly of introns or intergenic spacers and thus should fit the neutral variation model. Using the four-gamete criterion [43], we do not find intragenic recombination in the nuclear sequences among these three species. The data of the three plastid fragments were combined because the chloroplast genes are generally linked and no evidence of recombination between the three regions is found.

To run IMa2, one random haplotype per plant individual was chosen for the nuclear gene data sets, and the plastid data set was composed of sequences from the same plant individuals. This avoids bias but decreases the amount of information and thus leads to broader posterior distributions. The IS (Infinite Sites) model [44] of sequence evolution was chosen for the plastid locus, whereas, the HKY model [45] which allows for multiple substitutions was selected for the two nuclear loci because double mutations were found for a few polymorphic sites at both loci. The inheritance scalar was set to 1.0 for the nuclear and to 0.25 for the plastid loci, respectively.

To set upper bounds on the prior distributions of the parameters, we estimated for each of the three species the geometric means of the population mutation rate *4Nu *across all three loci using Watterson's estimator *θ *(per sequence not per site). The largest mean value was found with *A. asiatica*-2x (an estimate of *4Nu *= 9.8205), and this was used to set the upper bound on uniform prior for each of the three population demographic parameters: population size (*θ = *4*Nu*), splitting time (*t *= *Tu*, where *T *is the time in generations since the common ancestry, and it is of the same order of 4*N*) and migration rates (2*NM *= 4*Nu *× m/2). The priors were finally set as follows: the upper bound of population sizes *q *= 100, splitting times *t *= 5 and migration rates *m *= 2.0, respectively. We ran the Markov-chain Monte Carlo (MCMC) simulations with 1,000,000 burn-in steps and 20,000 genealogies sampled per locus. The analysis was done with 10 independent runs in the M mode, each using identical priors and 20 Metropolis-Coupled chains with different random number seeds. The genealogies sampled from the M mode runs were combined in an L mode run to build an estimate of the joint posterior probability of the parameters [26,27].

## Results

### Nuclear gene trees with allelic haplotype sequences

Amplification of the partial ncp*GS *and *SBP *genes produced a single clear band for each amplification. This and the results from earlier work (see "Methods") suggest sequences of the two nuclear loci obtained here each as belonging to a set of orthologs.

After eliminating some sequences likely containing PCR-recombination (about 10% of the total), 303 sequences (clones) of the ncp*GS *and 313 of the *SBP *gene from broadly the same 70 individuals of 29 populations belonging to *A. millefolium *agg. were used for the data analyses (Table 1). In addition, 56 ncp*GS *and 36 *SBP *sequences from five populations of three congeneric species outside the *A. millefolium *agg., *A. nobilis*-2x, *A. ligustica*-2x and *A. acuminata*-2x, were also analyzed here. The ncp*GS *alignment contains 918 nucleotide positions with sequence length varying from 795 to 861 bps. The *SBP *alignment contains 420 nucleotide positions with sequence length varying from 386 to 405 bps.

**Figure 1:**
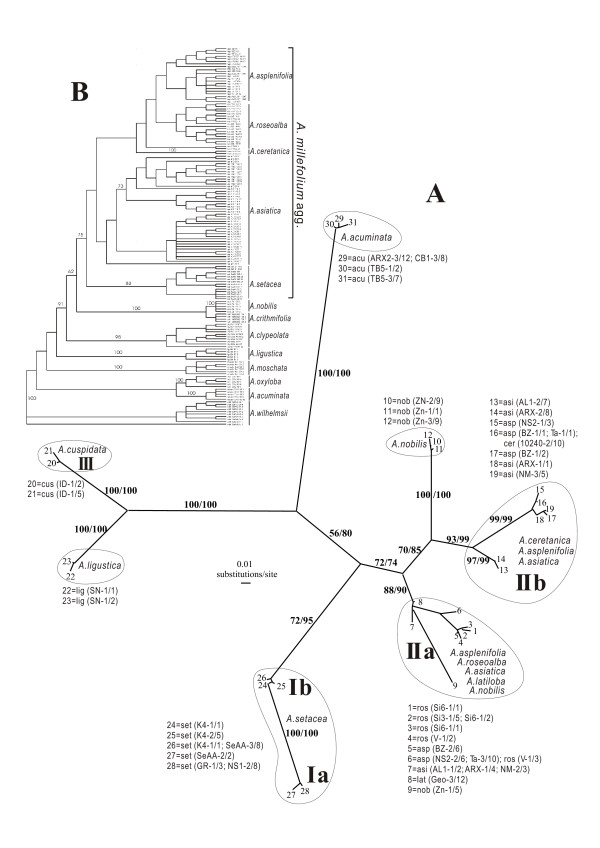
**The ncp*GS *gene tree and the AFLP tree**. **A**. Unrooted Neighbour Joining phylogram of 10 diploid *Achillea *species (seven of and three outside *A*. *millefolium *agg.) based on the ncp*GS *gene sequence data. The tree contains 31 ncp*GS *haplotypes generated from 186 clones (sequences) from 49 individuals of 20 populations. Bootstrap supports (> 50%) from both methods (NJ/MP) are shown next to the major branches. Labels of terminal branches are written as "taxon abbreviation (population code-number of individuals/number of clones)". For taxa abbreviations, see Table 1. **B**. The AFLP tree from a previous study (Guo *et al*., 2005 [10]) for comparison.

**Figure 2:**
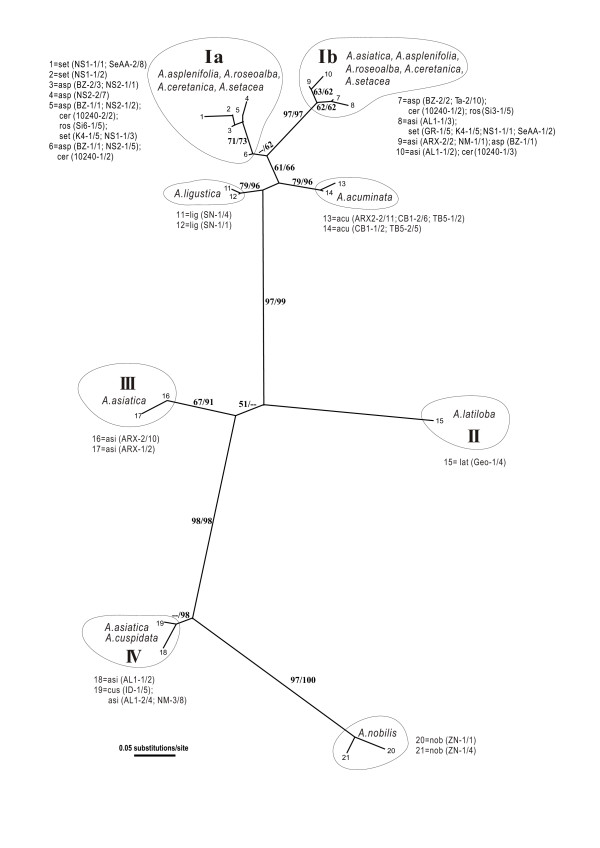
**Unrooted Neighbour Joining phylogram of 10 diploid *Achillea *species (seven of and three outside *A*. *millefolium *agg.) based on the *SBP *gene sequence data**. The tree contains 21 *SBP *haplotypes generated from 163 clones (sequences) from 35 individuals of 19 populations. Bootstrap supports (> 50%) from both methods (NJ/MP) are shown next to the major branches. Labels of terminal branches are written as "taxon abbreviation (population code-number of individuals/number of clones)". For taxa abbreviations, see Table 1.

Prior to the analyses of all the diploid and polyploid taxa, we present the gene trees at the diploid level first (Figures 1 &2).

The diploid-only ncp*GS *data come from seven species within and three outside *A. millefolium *agg.. They contain 31 haplotypes with 134 substitution sites from 186 sequences (Additional file 1: S-Figure 1). Out of the 134 polymorphic sites, 122 are in introns. Intragenic recombination among some samples is likely: A discordance in the alignment can be resolved by postulating a recombination in three of the four ncp*GS *haplotypes of *A. nobilis*-2x around the 89^th ^polymorphic site (Additional file 1: S-Figure 1). Another discordance can be resolved in the two haplotypes of *A. cuspidata*-2x (III in Figure 1A) by postulating a recombination around the 26^th ^polymorphic site between haplotype groups of *A. millefolium *agg. and of *A. ligustica*-2x (Additional file 1: S-Figure 1).

Figure 1A shows an unrooted Neighbour Joining phylogram based on the diploid-only ncp*GS *data. The topology of the MP tree on the same data set is broadly comparable, and thus only bootstrap values from the MP analysis are presented in Figure 1A. In this gene tree, haplotypes of *A. millefolium *agg. fall into two major groups, group I corresponding to *A. setacea*-2x, and group II those of the other Eurasian diploid species except *A. cuspidata*-2x. This relationship agrees with that from the previous AFLP analysis (Figure 1B inferred from Guo *et al*., 2005). In haplotype group II, both the subgroups IIa and IIb harbor sequences of *A. asplenifolia*-2x and *A. asiatiaca*-2x. This is not in line with the AFLP tree and is most likely due to retention of ancestral polymorphism or secondary contacts between the two species. *A. asplenifolia*-2x shares its diverged alleles with *A. ceretanica*-2x and *A. roseoalba*-2x, respectively, corresponding to the previous AFLP tree (Figure 1B). Sequences of *A. ceretanica*-2x (relict in the Eastern Pyrenees) only appear in IIb and those of *A. roseoalba*-2x (from the meadows in the S-Alps) only in IIa. The sequences of *A. cuspidata*-2x (in the Himalayas) appear distant to other members of *A. millefolium *agg. but close to *A. ligustica*-2x (Figure 1A). The single plant sample of *A. cuspidata*-2x was collected recently and thus was not included in the previous AFLP study. Clearly more samples of this species should be investigated. Out of the four haplotypes of *A. nobilis*-2x, one falls into clade IIa together with several members of *A. millefolium *agg., and three form a group between IIa and IIb. The latter can be explained by the intragenic recombination visible in the alignment (Additional file 1: S-Figure 1). Considering relationships of *A. nobilis *with *A. millefolium *agg., the ncp*GS *gene tree is neither congruent with the AFLP tree (Figure 1B) nor with the *SBP *tree (see below, Figure 2).

The diploid-only *SBP *data also include seven species within and three outside *A. millefolium *agg. They contain 21 haplotypes with 60 substitution sites based on 163 sequences. Out of the 60 polymorphic sites, 48 belong to the intron regions. The alignment of the *SBP *sequences does not show obvious intragenic recombination (Additional file 2: S-Figure 2). The topology of the NJ tree is broadly comparable with that of the MP tree and thus only bootstrap values from the MP analysis are presented (Figure 2). The *SBP *gene tree (Figure 2) is remarkably incongruent with the ncp*GS *tree (Figure 1A) and with the AFLP tree (Figure 1B). In Figure 2, haplotypes belonging to members of *A. millefolium *agg. do not group together. Some of *A. asiatica*-2x, all of the Caucasus *A. latiloba*-2x and the Himalayan *A. cuspidata*-2x (here defined as Asian types) are distantly related to the others of *A. millefolium *agg. Surprisingly, sequences of the C European *A. nobilis*-2x are close to the Asian type, whereas, haplotypes of the E Asian *A. acuminata*-2x are close to the major haplotype group of *A. millefolium *agg., which is mostly of the European members. We thus observe little sorting of ancestral polymorphisms of the *SBP *gene during the speciation processes of the diploid species of *Achillea*.

**Figure 3:**
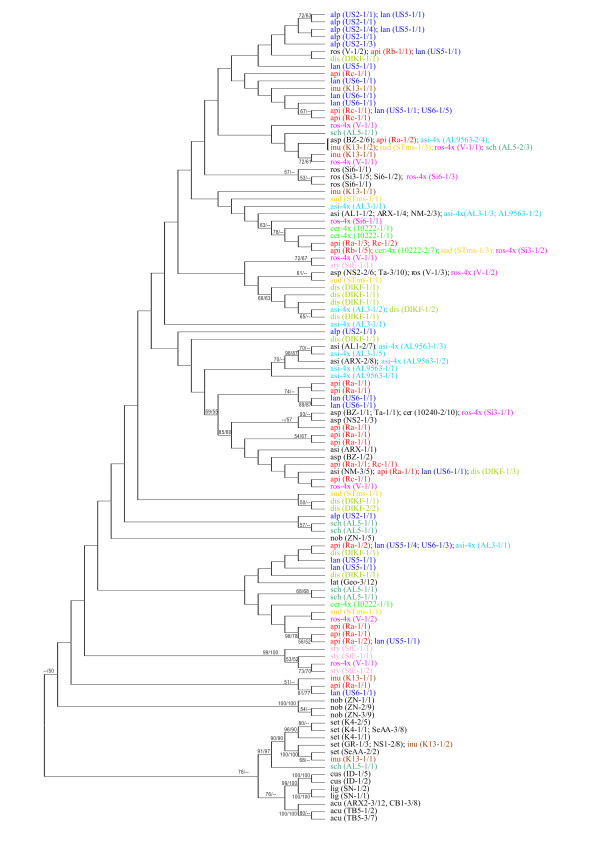
**Unrooted Neighbour Joining cladogram of the ncp*GS *gene of all the diploid and polyploid taxa analyzed in this study**. The tree contains 109 allelic haplotypes generated from 359 sequences with 155 substitution sites. Topology of the MP tree on the same data set is broadly comparable with that of the NJ tree. Bootstrap supports (> 50%) from NJ/MP analyses are shown next to the branches. Label of each terminal branch is written as "taxa abbreviation (population code-number of individuals/number of clones)". For taxa abbreviations, see Table 1. Diploid taxa are in black, polyploid taxa in different colours.

Figure 3 shows the ncp*GS *gene tree (unrooted NJ tree) of all the diploid and polyploid taxa of *A. millefolium *agg. and of three congeneric diploid speices. It is based on 110 haplotypes generated from 359 sequences with 155 polymorphic substitution sites. In Figure 3, alleles of each polyploid individual, population or taxon (marked in different colors) scatter among haplotypes of different diploid species (all in black letters) of *A. millefolium *agg. except *A. cuspidata*-2x. *A. roseoalba*-4x and *A. asiatica*-4x share some of their alleles with their diploid cytotypes, respectively, whereas *A. ceretanica*-4x is quite differentiated from *A. ceretanica*-2x. Alleles from the tetraploid *A. borealis *var. *alpicola *and var. *lanulosa-*4x in N America, the tetraploid *A. asiatica*-4x in C Asia and the hexaploid *A. millefolium *subsp. *apiculata*-6x in NE Europe are more often associated with each other than with those of other polyploid taxa. Only the Ukrainian *A. inundata*-4x and the C Asian *A. schmacovii*-6x share nuclear haplotypes with *A. setacea*-2x.

The date set of *SBP *gene of all the diploids and polyploids contains 68 haplotypes generated from 349 sequences with 68 polymorphic substitution sites. Due to the severe conflicts between the *SBP *gene tree and the species tree inferred from the AFLP and morphological data, sequences of this gene are not suitable for the phylogenetic inference, but could provide some clues about the progenitors of the polyploid taxa. We therefore only present the *SBP *gene tree of all the diploid and polyploid samples in the supplementary materials as Additional file 3: S-Figure 3.

### Phylogenetic networks based on plastid haplotypes

Thirty populations with broadly the same 70 individuals analyzed with the nuclear genes were sequenced at three plastid loci, *trnH-psbA*, *trnC-ycb6 *and *rpl*16. The length variation and number of polymorphic sites of each fragment are listed in Table 2.

**Table 2:** Sequence characters of the analyzed cpDNA non-coding regions

	*trnH-psbA*	*trnC-ycf6 *(incl. partial *ycf6-psbM*)	*rpL16 *(for diploid taxa only)	All three fragments combined for diploid taxa only	*trnH-psbA *and *trnC-ycf6 *combined for all taxa
Length	424-445 bp	537-564 bp	849-876 bp	1814-1842 bp	960-989 bp
Number of haplotypes	13	18	8	13	26
Number of variable sites	11	18	9	26	29
Number of indels (length in base pairs) *	3 (5; 1; 21)	5 (1; 6; 1; 21)	4 (5; 5; 1; 22)	8 (1; 6; 5; 1; 5; 5; 1; 22)	8 (1; 6; 1; 6; 21; 5; 1; 21)

* Indels were coded as binary characters (0/1 = A/C) using software *GapCoder *[37]

**Figure 4:**
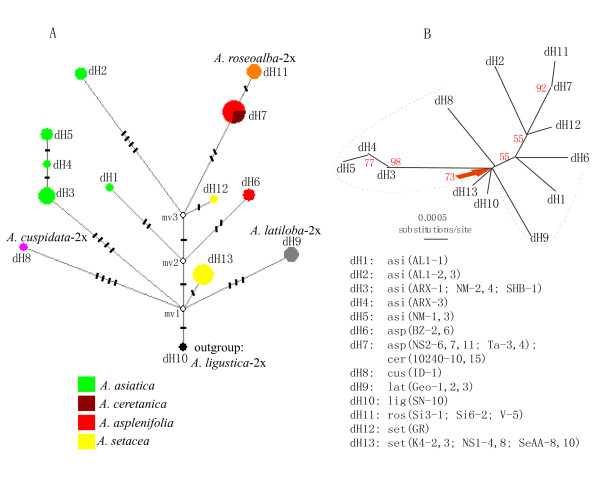
**Medium-Joining Network (**A**) and unrooted NJ tree (**B**) of 13 cpDNA haplotypes (dH1-13) across 34 individuals of 18 populations of the diploid species of *A. millefolium *agg. and the congeneric *A. ligustica*-2x.** These haplotypes are generated from three noncoding cpDNA regions, *trnH-psbA*, *trnC-ycf6 *and *rpL16*. Short bars on branches of the network indicate the number of variable sites (incl. gap polymorphisms, see Table 2). Red numbers next to the branches of the NJ tree are bootstrap values. Plant individuals are labelled as "taxa abbreviation (population code-individual identity No.)". For taxa abbreviations, see Table 1.

For the diploid members of *A. millefolium *agg. together with their sister species *A. ligustica*, three plastid fragments from 18 populations and 34 individuals generated a combined matrix with 1855 (varying from 1814 to 1842) nucleotide positions and 26 variable sites. Out of the 26 variable sites, 18 are substitution sites and 8 are indels (Table 2). The polymorphic sites allow the identification of 13 plastid haplotypes named as dH1-13, where "d" stands for diploids to be distinguished from those used for the diploid- polyploid combined data as described below. As shown in Figure 4, polymorphic plastid haplotypes are found within each of the three relatively widespread species *A. setacea*-2x, *A. asplenifolia*-2x and *A. asiatica*-2x. Furthermore, distribution of the plastid polymorphism is not even among the diploid species. Among the three widespread species, the European *A. setacea *and *A. asplenifolia *each harbours a relatively frequent haplotype, dH7 and dH13, respectively, whereas *A. asiatica*-2x in Asia exhibits 5 haplotypes.

**Figure 5:**
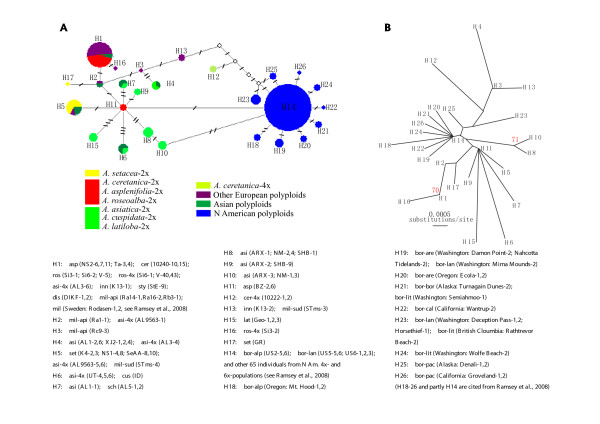
**Medium-Joining Network (**A**) and unrooted NJ tree (**B**) of 26 cpDNA haplotypes (H1-26) across the N Hemisphere populations of the diploid and polyploid taxa of *A. millefolium *agg**. The data are based on sequences of two cpDNA noncoding regions, *trnH-psbA *and *trnC-ycf6*. Short bars on branches of the network indicate the number of variable sites (incl. gap polymorphisms, see Table 2). Red numbers next to the branches of the NJ tree are bootstrap values. Plant individuals are labelled as "taxa abbreviation (population code-individual identity No.)". For taxa abbreviations, see Table 1.

To illustrate the formation of the polyploids and their worldwide migration, we incorporated the plastid *trnH-psbA *and *trnC-ycf6 *sequences obtained by Ramsey *et al*. [12] from the 4x and 6x N American *A. borealis *populations available from the NCBI data base (accession No. EU128982-129456) into our diploid-polyploid combined data. Our *rpL*16 intron sequences were left out because this gene was not sequenced for the populations analyzed by Ramsey *et al*. [12]. The resulting data matrix thus contains 156 individuals and 1024 nucleotide positions (sequences varying from 960 to 989 bps in length). This allows identification of 26 haplotypes (H1-26) on the basis of 29 variable characters, of which 21 are nucleotide substitutions and 8 are indels (Table 2). Relationships among these haplotypes are shown in Figure 5.

H1 is the most frequent haplotype in Eurasia. It is shared by three out of four European diploid species and is spread among most of the Eurasian polyploid taxa at all ploidy levels (Figure 5). The rare H11 in Europe (only in *A asplenifolia*-2x) is related to the Eurasian H5, to most of the Asian types H 6-9 &15, and even to H14 which is the most frequent in N America. The European polyploid specific H3 (in *A. millefolium *subsp. *apiculata*-6x) and H13 (*A. millefolium *subsp. *sudetica*-6x &*A. inundata*-4x) are directly or indirectly related to H4 found in *A. asiatica*-2x & -4x. The N American frequent H14 has been derived most probably from the E Asian H10 and evidently gave rise within N America to several rare and more local haplotypes (H18-22, 24, 26).

The 26 plastid haplotypes are mapped associated with the general distribution of populations studied here in the Additional file 4: S-Figure 4.

### Demography of major diploid lineages

**Figure 6:**
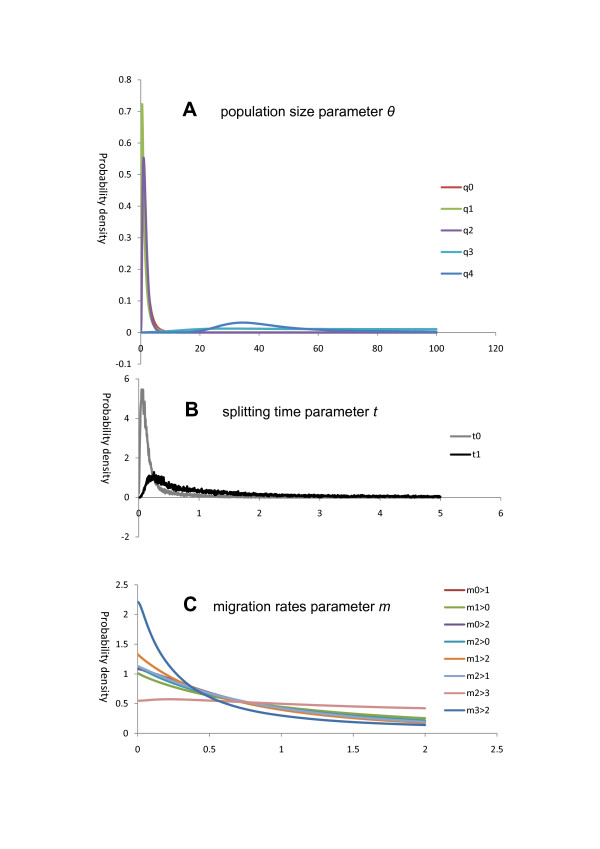
**Marginal posterior probability density for each of the population size (*θ*, written as *q *in panel A), splitting time (*t*) and migration rates (*m*) parameters under a multi-population model (IMa2) for three major diploid species *A. asiatica*-2x, *A. asplenifolia*-2x and *A. setacea*-2x**. In panel A & C, the number "0" stands for *A. asiatica*-2x, "1" for *A. asplenifolia*-2x, "2" for *A. setacea*-2x, "3" for the common ancestral population of *A. asiatica*-2x and *A. asplenifolia*-2x, and "4" for the ancestral population common to all three species. In panel B, *t*0 refers to the splitting time of *A. asiatica*-2x and *A. asplenifolia*-2x, and *t*1 represents the time when *A. setacea*-2x split from the ancestor of *A. asplenifolia*-2x and *A. asiatica*-2x. In panel C, " > " indicates direction of migrants, e.g., m0 > 1 refers to migration from 0 (*A. asiatica*) to 1 (*A. asplenifolia*). Each curve is the sum of 10 curves from the analysis of 10 independent MCMC simulations.

Figure 6 shows the plots of the joint posterior probability distributions of each of the demographic parameters. Panel A of Figure 6 indicates sharp peaks close to zero for the effective population sizes of the three species after their splits, i.e., *q*0 (of *A. asiatica-*2x), *q*1 (of *A. asplenifolia*-2x) and *q*2 (of *A. setacea*-2x). The speciation times are rather short (panel B), especially *t*0, i.e., the split between *A. asiatica-*2x and *A. asplenifolia*-2x, is inferred to have occurred very recently. The speciation time *t*1 is bounded away from zero, which corroborates our assumption of *A. setacea *splitting first. We note that these inferred parameters are consistent with the data. While the effective population size *q*0 is relatively small, *t*0 is very short, such that their ratio *q*0/*t*0 is still relatively large, which corresponds to relatively little drift and thus the relatively large diversity in *A. asiatica-*2x compared to the other two species. Drift is higher in the other species: The effective population size within *A. asplenifolia*-2x, i.e., *q*1, is estimated to be even smaller than *q*0 of *A. asiatica-*2x, which combines with *t*0 to explain the reduced diversity in this species compared to *A. asiatica-*2x. The estimate of effective population size in *A. setacea*-2x, *q*2, is about the same as *q*0 in *A. asiatica-*2x, however the time since the split, i.e., *t*1 is longer, such that the product *q*2/*t*1 is smaller than *q*0/*t*0 in *A. asiatica-*2x. Note that the combination of short times and small effective population sizes means that the species are mainly differentiated by drift and not by mutations.

In contrast, all ancestral population sizes are large compared to the current ones: *q*3, the ancestral effective population size before the split of *A. asiatica*-2x and *A. asplenifolia*-2x, and *q*4, the ancestral population common to all three species are bounded away from zero. The distributions of the migration parameters (panel C) are broad and quite similar to the equal prior distributions, such that we conclude that there is little information for inference of these parameters in the data. Setting wider maximum priors for *m *did not result in convergence. We note that differentiation of subdivided populations that exchange few migrants and populations that split and afterwards do not exchange migrants lead to rather similar molecular variation patterns. Therefore, differentiating between migration and drift and temporal population subdivision is difficult. This likely explains the broad posterior distribution of the inferred migration parameters.

## Discussion

Within the *Achillea millefolium *aggregate, diploid species are usually well separated and their relationships conform to a binary bifurcating tree according to the AFLP data [10]. At the polyploid level, species are difficult to define either by morphology or by molecular data. Previous AFLP data show the polyploid taxa mostly polytomic and polyphyletic [10,11]. With the haplotype sequence data from both nuclear and plastid genomes available here, we try to infer population history and demography of the diploid species and to untangle the complex relationships among the polyploids.

### Gene trees versus species trees of the diploids

The gene trees from the nuclear and the plastid loci of the diploid populations of *A. millefolium *agg. are incongruent with each other and with the previous AFLP species tree. None of the gene trees corresponds well with the morphological and cytogenetical differentiation of the diploid species, whereas the AFLP tree does (Figures 1, 2 &4; [10]). For the ncp*GS *gene, haplotypes of each of the widespread *A. asplenifolia*-2x and *A. asiatica*-2x belong to two groups, IIa and IIb (Figure 1A). Relevant ncp*GS *sequences indicate intragenic recombination (Additional file 1: S-Figure 1). The *SBP *gene tree conflicts more severely with the AFLP tree and the species delimitation (Figure 2). Even the plastid sequences show polymorphic haplotypes within each of the three widespread species *A. setacea*-2x, *A. asplenifolia*-2x and *A. asiatica*-2x (Figure 4). As the phylogenetic relationships of these three species indicated by the AFLP tree (Figure 1B) are in line with the morphological and biogeographical information, we suggest that the gene tree incongruence as well as their discordance with the inferred species tree (asserted by the AFLP tree) are due to a lack of sorting of ancestral polymorphic alleles and/or due to introgression after the split of the species. Assuming neutrality, retention of ancestral polymorphism is likely if speciation is fast relative to drift within the populations [41]. The quantitative results from the IM model (Ima2 program [27]) show large ancestral effective population sizes, short splitting time between them and some migration. This corroborates our inference from the discordant nuclear gene trees and suggests rapid speciation and/or occasional exchange of migrants at the diploid level within *A. millefolium *agg..

The same pattern apparently also characterizes the more distantly related congeneric species, e.g., *A. nobilis*-2x, *A. ligustica*-2x and *A. acuminata*-2x (Figures 1 &2). In *A. nobilis*, a diploid species relatively close to *A. millefolim *agg., we find intra-locus recombination in the ncp*GS *gene, indicating its hybrid origin or introgression involving a diploid donor from *A. millefolim *agg. (Additional file 1: S-Figure 1; Figure 1A).

### Phylogeography and rapid speciation

Throughout the N Hemisphere, gene flow between divergent lineages through periods of climate changes has shaped the extant geographical distribution and patterns of genetic variation of many plant species [46-51]. Rapid speciation resulted from post-glacial hybrid contacts and polyploidy during population migration have often been reported and documented. For example, Brysting *et al*. [34,52] has untangled the complex history of the polyploid *Cerastium alpinum *group in the circumpolar arctic flora. Complexity is also evident in the evolutionary radiation of *A. millefolium *agg [10-12].

The diploid taxa of *A. millefolium *agg. are limited to Eurasia, following an eco-geographical vicariance pattern but appear to be disjunctive and regressive evidently under the pressure of their more expansive polyploid descendants: *A. ceretanica*-2x, a relict endemic in subalpine grassland of the E Pyrenees; *A. asplenifolia*, an endemic relict of humid lowland grasslands in the Pannonian plains from Bulgaria, Hungaria, E Austria and the adjacent Czech Rep.; *A. roseoalba*-2x, a variable taxon of mesic forest margins and anthropogenous meadows in the geologically recent N Italian plains and foothills with populations in adjacent Switzerland, Austria, and locally even in Bavaria and Slovenia where it is in close contact with *A. asplenifolia*-2x; *A. setacea *in steppes from SW Asia and SE Europe to continental areas of C Europe and the Alps; *A. latiloba*, today a subalpine relict in the mountains of NE Turkey and SW Georgia; *A. asiatica*-2x in montane to alpine grassland from the Altai to Mongolia and N China; and *A. cuspidata*-2x, a relict in the W Himalayas.

The previous AFLP data [10] indicate that *A. setacea*-2x branches early within the *A. millefolium *agg. The present sequence data partly show compatible patterns of relationships, i.e., the European species *A. ceretanica*-2x, *A. roseoalba*-2x and *A. asplenifolia*-2x are more closely related to each other and to the Asian *A. asiatica*-2x than to *A. setacea*-2x (Figures 1 &4). The morphology, distribution and habitat preferences suggest that *A. roseoalba*-2x might have originated from introgression into *A. asplenifolia *possibly by the *A. ceretanica*-like populations [15,53]. Compared to its European sister species, *A. asiatica*-2x represents a wide geographic extension of *A. millefolium *agg. into C and E Asia and harbors the richest plastid variation among all the diploid taxa (Figure 4). It could have survived the cold periods of the late Pleistocene in refugia not far from its present occurrence in or near the Asian mountain areas. In spite of the large distributional range, we find little evidence for isolation by distance: The easternmost *A. asiatica*-2x and the westernmost *A. ceretanica*-2x share as much genetic variation as each with the geographically intermediate *A. asplenifolia*-2x and *A. roseoalba*-2x (Figures 1 &2).

In contrast to the diploid species that show phylogenetic structuring and are limited to Eurasia, the polyploid taxa of *A. millefolium *agg. exhibit practically continuous and interrelated relationships and have extended their range to N America. With the available nuclear ncp*GS *and *SPB *sequence data alone, it is often hard to decide whether a polyploid taxon studied here is auto- or allopolyploid because even the diploids share polymorphic alleles likely due to incomplete lineage sorting and/or secondary introgression. Only combined with the previous AFLP profiles [10], the autopolyploid nature of some may become clear, but most are influenced by hybridization.

In Europe, *A. ceretanica*-4x in central France exhibits some ncp*GS *correspondence (Figure 3) with *A. roseoalba*-4x and AFLP affinities with *A. ceretanica*-2x [10] but otherwise little affiliation with its diploid cytotype. In populations of *A. roseoalba*-2x, 4x-individuals occur with a new plastid haplotype (H16 in Figure 5) and corresponding ncp*GS *alleles (Figure 3). *A. styriaca*-4x, an endemic from E Austria and the Czech Rep., is ecologically distinct but shares ncp*GS *and *SBP *alleles with *A. roseoalba*-4x and the plastid haplotype H1 with *A. asplenifolia*-2x and so on. *A. setacea*-2x and *A. asplenifolia*-2x have been involved in the origin of the widespread and expansive C to E European allotetraploid *A. collina*-4x [10,19]. All the 4x-taxa mentioned above are connected by occasional intermediates, can be easily hybridized in crossing experiments and produce viable progeny with more or less normal meioses [54,55]. Further to the east in Ukraine, *A. inundata*-4x is linked to *A. setacea-*2x and also to *A. asplenifolia*-2x *etc*. (Figures 3 &5).

The diverse 4x-cytotypes from C and E Asia are provisionally called *A. asiatica-*4x. They are particularly linked to *A. asiatica*-2x, but also to *A. cuspidata-*2x (Figure 5; Additional file 3: S-Figure 3). Plastid H1 and H5 also demonstrate links of *A. asiatica-*4x with the European 2x- and 4x-taxa (Figure 5). How complex the relationships of higher polyploids in *A. millefolium *agg. are is shown by the recently described Altai endemic *A. schmakovii*-6x: it shares nuclear haplotypes with *A. setacea*-2x, *A. asplenifolia*-2x, *A. inundata*-4x, *A. asiatica*-4x and has the rare plastid haplotype H7 from *A. asiatica*-2x.

The Pleistocene extension of *A. millefolium *agg. into N America [12] with populations called *A. borealis *s.lat. and two main cytotypes (4x and 6x [6]) is of particular interest. They have developed ecotypes in most habitats from the sand dunes of the Pacific to the peaks of Rocky Mts. and the East Coast forests. Their rapid adaptive differentiation has been well documented by Clausen *et al*. [6] and Ramsey *et al*. [12]. The molecular genetic data from Guo *et al*. [10,11] and Ramsey *et al*. [12] have shown that all these native N American populations differ from those in Eurasia, are monophyletic and most likely linked to *A. asiatica*-2x/4x (called *A. millefolium *var. *manshurica *Kit. [12]), but can not be resolved in more details. In the ncp*GS *and *SBP *gene trees (Figure 3; Additional file 3: S-Figure 3), they share alleles with *A. asiatica*-2x and -4x, but also with *A. millefolium*-6x (*A. apiculata*) in subarctic Russia and even with *A. inundata*-4x, *A. ceretanica*-4x and *A. asplenifolia-*2x. Considering plastid haplotypes, the most frequent H14 is likely the ancestral, from which H18-H22, 24, 26 have originated (Figure 5). All these and additional fossil data [12,56,57] support the assumption that the ancestors of the N American populations might have survived in NE Asian/Alaskan refugia ("Beringia") during the middle or late Pleistocene cold periods [12,49]. Apart from the westward extension through Siberia to subarctic Europe (*A. millefolium*-6x*/A. apiculata*-6x), their main migrations might have been east- and southward into N America. There, without the competition of closely related taxa, they underwent a radiation and formed the 4x and 6x racial complex of *A. borealis *s.lat.. This can be regarded quasi as a model for the early phase of the eco-geographical radiation of *A. millefolium *agg. in SE Europe and adjacent SW Asia, and a second phase with *A. asiatica-*2x+4x, *A. alpina*-4x and *A. schmakovii*-6x in C and E Asia [10,11,15,58,59].

## Conclusions

Nuclear and plastid haplotype data analyzed in this study suggest rapid diploid and polyploid speciation in the temperate N Hemisphere *Achillea *species, especially those within *A. millefolium *agg.. Hybridization and polyploidy seem to have promoted the recent lineage radiation, shaped the concurrent patterns of genetic variation, and contributed to the wide distribution of this species complex.

The sequence data from two nuclear genes and chloroplast DNA employed in this study result in incongruent trees, obviously due to lack of lineage sorting and/or secondary hybridization, and thus cannot resolve the species phylogeny. This lack of lineage sorting apparently extends to other congeneric taxa. To date, the AFLP tree [10] is the only molecular tree that can be brought into accordance with species delimitations, morphology and traditional taxonomy of *A. millefolium *agg.. This is likely due to the averaging effect of the genome-wide sampling of AFLP polymorphism.

With little lineage sorting and frequent gene flow, the species tree can only be recovered using data from many unlinked DNA regions. Despite the development of new techniques such as the RAD tag technology [60], traditional molecular methods, such as AFLP genome screening, are still useful for non-model species, especially if complemented by likelihood-based Bayesian analytical methods [61]. On the other hand, even if sequence data from the nuclear and chloroplast genomes can not override the AFLP data in resolving recent species divergence, they do help to understand population demography and speciation processes, and to demonstrate that shared ancestral polymorphisms are more common than fixed alleles in young radiating species. Therefore, for the phylogeographic surveys of non-model organisms, we advocate the use of DNA polymorphisms from multiple unlinked loci, *e.g*., AFLP markers, combined with sequence data from some single genes, as such a combination appears useful and cost and time efficient.

## Authors' contributions

YPG conceived the project, collected part of the plant samples, conducted the data analysis and led the writing; SZW performed the lab work and collected the sequence data; CV helped on conceiving the ideas and manuscript drafting; and FE collected and identified most of the plant materials, contributed phylogeographic aspects and helped with manuscript writing. All authors read and approved the final manuscript.

## Supplementary Material

Additional file 1 **The aligned polymorphic sites among 31 haplotypes of the sequenced ncp*GS *gene**. These haplotypes are generated from 134 substitution sites among 186 clones (sequences) from 49 individuals of 20 populations of 10 diploid *Achillea *species (seven within and three outside *A. millefolium *agg.). Abbreviations of the species: (1) of *A. millefolium *agg.: asi = *A. asiatica*, asp = *A. asplenifolia*, cer = *A. ceretanica*, cus = *A. cuspidate*, lat = *A. latiloba*, ros = *A. roseoalba *and set = *A. setacea*; (2) of other species: acu = *A. acuminata*, lig = *A. ligustica *and nob *= A. nobilis*. Title of each haplotype sequence includes: abbreviation of species (number of populations/individuals/clones). Note: three haplotype of *A. nobilis *(nob) except the first one contain a recombination around the 89^th ^polymorphic site between two or three haplotype groups of *A. millefolium *agg. (one above *A. nobilis*, and the others below). The sequences of *A. cuspidate *(cus) seems also containing a recombination around the 26^th ^polymorphic site between those of *A. ligustica *(lig_SN) and some of *A. millefolium *agg. (e.g., several sequences at the top of the matrix). Click here for file

Additional file 2 **The aligned polymorphic sites among 21 *SBP *haplotypes**. These haplotypes are generated from 60 substitution sites among 163 clones (sequences) from 35 individuals of 19 populations of 10 diploid *Achillea *species (seven within and three outside *A. millefolium *agg.). Abbreviations of the species: (1) of *A. millefolium *agg.: asi = *A. asiatica*, asp = *A. asplenifolia*, cer = *A. ceretanica*, cus = *A. cuspidate*, lat = *A. latiloba*, ros = *A. roseoalba *and set = *A. setacea*; (2) of other species: acu = *A. acuminata*, lig = *A. ligustica *and nob *= A. nobilis*. Title of each haplotype sequence includes: abbreviation of species (number of populations/individuals/clones). Click here for file

Additional file 3 **Unrooted Neighbour Joining cladogram of the *SBP *gene of all the diploid and polyploid taxa analyzed in this study**. The tree contains 68 allelic haplotypes generated from 349 sequences with 68 substitution sites. Topology of the MP tree on the same data set is broadly comparable with that of the NJ tree. Bootstrap supports (> 50%) from NJ/MP analyses are shown next to the branches. Label of each terminal branch is written as "taxa abbreviation (population code-number of individuals/number of clones)". For taxa abbreviations, see Table 1. Diploid taxa are in black, polyploid taxa in different colors. Click here for file

Additional file 4 **Map showing the approximate distribution the 26 plastid haplotypes (H1-26) recognized across the temperate N Hemisphere**. Pies indicate the proportion of haplotypes registered for individual taxa/cytotypes and the size of each pie correlated to the sample size from their generalized sampling areas. Click here for file

## References

[B1] Grant V Plant Speciation 1981 New York, Columbia University Press

[B2] Vijverberg K Kuperus P Breeuwer JAJ Bachmann K Incipient adaptive radiation of New Zealand and Australian *Microseris *(Asteraceae): an amplified fragment length polymorphism (AFLP) study J Evol Biol 2000 13 997 1008 10.1046/j.1420-9101.2000.00241.x

[B3] Baldwin BG Carlquist S, Baldwin BG, Carr GD A phylogenetic perspective on the origin and evolution of Madiinae Tarweeds & Silverswords, Evolution of the Madiinae (Asteraceae) 2003 St. Louis: Missouri Botanical Garden Press 193 22820715636

[B4] Chase MW Knapp S Cox AV Clarkson JJ Butsko Y Joseph J Savolainen V Parokonny AS Molecular systematics, GISH and the origin of hybrid taxa in *Nicotiana *(Solanaceae) Ann Bot 2003 92 107 127 10.1093/aob/mcg087 12824072 PMC4243627

[B5] Soltis DE Soltis PS Tate JA Advances in the study of polyploidy since plant speciation New Phytol 2003 161 173 191 10.1046/j.1469-8137.2003.00948.x

[B6] Clausen J Keck D Hiesey WM Experimental studies on the nature of species. III. Environmental responses of climatic races of *Achillea* Carnegie Institute Washington Publ 1948 581

[B7] Anderson E Stebbins GL Hybridization as an evolutionary stimulus Evolution 1954 8 378 388 10.2307/2405784

[B8] Schemske DW Population structure and local selection in *Impatiens pallida *(Balsaminaceae), a selfing annual Evolution 1984 38 817 832 10.2307/2408393 28555822

[B9] Linhart YB Grant MC Evolutionary significance of local genetic differentiation in plants Ann Rev Ecol Syst 1996 27 237 277 10.1146/annurev.ecolsys.27.1.237

[B10] Guo Y-P Saukel J Mittermayr R Ehrendorfer F AFLP analyses demonstrate genetic divergence, hybridization, and multiple polyploidization in the evolution of *Achillea *(Asteraceae-Anthemideae) New Phytol 2005 166 273 290 10.1111/j.1469-8137.2005.01315.x 15760370

[B11] Guo Y-P Saukel J Ehrendorfer F AFLP trees versus scatterplots: evolution and phylogeography of the polyploid complex *Achillea **millefolium *agg. (Asteraceae) Taxon 2008 57 153 169

[B12] Ramsey J Robertson A Husband B Conti E Rapid adaptive divergence in New World *Achillea*, an autopolyploid complex of ecological races Evolution 2008 62 639 653 10.1111/j.1558-5646.2007.00264.x 18039326

[B13] Ramsey J Polyploidy and ecological adaptation in wild yarrow Proc Natl Sci USA 2011 108 7096 7101 10.1073/pnas.1016631108 PMC3084070 21402904

[B14] Ehrendorfer F Cytology of *Achillea *hybrids Carnegie Institute Washington Year Book 1952 51 124 125

[B15] Ehrendorfer F Differentiation-hybridization cycles and polyploidy in *Achillea* Cold Spring Harbor Symp Quant Biol 1959 24 141 152 13819584 10.1101/sqb.1959.024.01.014

[B16] Ehrendorfer F New chromosome numbers and remarks on the *Achillea millefolium *polyploid complex in North America Oesterr Bot Z 1973 122 133 143 10.1007/BF01624798

[B17] Hiesey WM Nobs MA Genetic and transplant studies on contrasting species and ecological races of the *Achillea millefolium *complex Bot Gaz 1970 131 245 259 10.1086/336539

[B18] Tyrl RJ Origin and distribution of polyploid *Achillea *(Compositae) in western North America Brittonia 1975 27 187 196 10.2307/2805480

[B19] Ma J-X Li Y-N Vogl C Ehrendorfer F Guo Y-P Allopolyploid speciation and ongoing backcrossing between diploid progenitor and tetraploid progeny lineages in the *Achillea millefolium *species complex: Analyses of single-copy nuclear genes and genomic AFLP BMC Evol Biol 2010 10 100 10.1186/1471-2148-10-100 20388203 PMC2873412

[B20] Slotte T Huang H Lascoux M Ceplitis A Polyploid speciation did not confer instant reproductive isolation in *Capsella *(Brassicaceae) Mol Biol Evol 2008 25 1472 1481 10.1093/molbev/msn092 18417485

[B21] Strasburg JL Rieseberg LH Molecular demographic history of the annual sunflowers *Helianthus annuus *and *H. Petiolaris*--large effective population sizes and rates of long-term gene flow Evolution 2008 62 1936 1950 10.1111/j.1558-5646.2008.00415.x 18462213 PMC2601659

[B22] Slowinski JB Page RDM How should species phylogenies be inferred from sequence data? Syst Biol 1999 48 814 825 10.1080/106351599260030 12066300

[B23] Belfiore NM Liu L Moritz C Multilocus phylogenetics of a rapid radiation in the genus *Thomomys *(Rodentia: Geomyidae) Syst Biol 2008 57 294 310 10.1080/10635150802044011 18432550

[B24] Edwards SV Is a new and general theory of molecular systematics emerging? Evolution 2009 63 1 19 10.1111/j.1558-5646.2008.00549.x 19146594

[B25] Degnan JH Rosenberg NA Gene tree discordance, phylogenetic inference and the multispecies coalescent Trends Ecol Evol 2009 24 332 340 10.1016/j.tree.2009.01.009 19307040

[B26] Hey J The divergence of chimpanzee species and subspecies as revealed in multipopulation isolation-with-migration analyses Mol Biol Evol 2010 27 921 933 10.1093/molbev/msp298 19955478 PMC2877540

[B27] Hey J Isolation with migration models for more than two populations Mol Biol Evol 2010 27 905 920 10.1093/molbev/msp296 19955477 PMC2877539

[B28] Temsch EM Greilhuber J Genome size variation in *Arachis hypogaea *and *A. monticola *re-evaluated Genome 2000 43 449 451 10902707

[B29] Suda J Krahulcová A Trávnícek P Krahulec F Ploidy level versus DNA ploidy level: An appeal for consistent terminology Taxon 2006 55 447 450 10.2307/25065591

[B30] Doyle JJ Doyle JL A rapid DNA isolation procedure for small quantities of fresh leaf tissue Phytochem Bull 1987 19 11 15

[B31] Emshwiller E Doyle JJ Chloroplast-expressed glutamine synthetase (ncp*GS*): Potential utility for phylogenetic studies with an example from *Oxalis *(Oxalidaceae) Mol Phylogenet Evol 1999 12 310 319 10.1006/mpev.1999.0613 10413625

[B32] Chapman MA Chang JC Weisman D Kesseli RV Burke JM Universal markers for comparative mapping and phylogenetic analysis in the Asteraceae (Compositae) Theor Appl Genet 2007 115 747 755 10.1007/s00122-007-0605-2 17634914

[B33] Shaw J Lickey EB Beck JT Farmer SB Liu W Miller J Siripun KC Winder CT Schilling EE Small RL The tortoise and the hare II: Relative utility of 21 noncoding chloroplast DNA sequences for phylogenetic analysis Am J Bot 2005 92 142 166 10.3732/ajb.92.1.142 21652394

[B34] Brysting AK Oxelman B Huber KT Moulton V Brochmann C Untangling complex histories of genome mergings in high polyploids Syst Biol 2007 56 467 476 10.1080/10635150701424553 17562470

[B35] Cronn R Cedroni M Haselkorn T Grover C Wendel JF PCR-mediated recombination in amplification products derived from polyploid cotton Theor Appl Genet 2002 104 482 489 10.1007/s001220100741 12582722

[B36] Wu L Tang T Zhou R Shi S PCR-mediated recombination of the amplification products of the *Hibiscus Tiliaceus *cytosolic glyceraldehyde-3-phosphate dehydrogenase gene J Biochem Mol Biol 2007 40 172 179 10.5483/BMBRep.2007.40.2.172 17394766

[B37] Young ND Healy J Gapcoder automates the use of indel characters in phylogenetic analysis BMC Bioinformatics 2003 4 6 10.1186/1471-2105-4-6 12689349 PMC153505

[B38] Kimura M A simple method for estimating evolutionary rates of base substitutions through comparative studies of nucleotide sequences J Mol Evol 1980 16 111 120 10.1007/BF01731581 7463489

[B39] Bandelt HJ Forster P Roehl A Median-joining networks for inferring intraspecific phylogenies Mol Biol Evol 1999 16 37 48 10331250 10.1093/oxfordjournals.molbev.a026036

[B40] Clark AG Neutral behavior of shared polymorphism Proc Natl Acad Sci USA 1997 94 7730 7734 10.1073/pnas.94.15.7730 9223256 PMC33687

[B41] Nielsen R Beaumont MA Statistical inferences in phylogeography Mol Ecol 2009 18 1034 1047 10.1111/j.1365-294X.2008.04059.x 19207258

[B42] Hey J Nielsen R Multilocus methods for estimating population sizes, migration rates and divergence time, with applications to the divergence of *Drosophila pseudoobscura *and *D. persimilis* Genetics 2004 167 747 760 10.1534/genetics.103.024182 15238526 PMC1470901

[B43] Hudson RR Kaplan NL Statistical properties of the number of recombination events in the history of a sample of DNA sequences Genetics 1985 111 147 164 4029609 10.1093/genetics/111.1.147 PMC1202594

[B44] Kimura M The number of heterozygous nucleotide sites maintained in a finite population due to steady flux of mutations Genetics 1969 61 893 903 5364968 10.1093/genetics/61.4.893 PMC1212250

[B45] Hasegawa M Kishino H Yano T Dating of the human-ape splitting by a molecular clock of mitochondrial DNA J Mol Evol 1985 22 160 174 10.1007/BF02101694 3934395

[B46] Comes HP Kadereit JW The effect of quaternary climatic changes on plant distribution and evolution Trends Plant Sci 1998 3 432 438 10.1016/S1360-1385(98)01327-2

[B47] Hewitt GM Post-glacial re-colonization of European biota Biol J Linn Soc 1999 68 87 112 10.1111/j.1095-8312.1999.tb01160.x

[B48] Hewitt GM The genetic legacy of the quaternary ice ages Nature 2000 405 907 913 10.1038/35016000 10879524

[B49] Hewitt GM Speciation, hybrid zones and phylogeography--or seeing genes in space and time Mol Ecol 2001 10 537 549 11298967 10.1046/j.1365-294x.2001.01202.x

[B50] Abbott RJ Smith LC Milne RI Crawford RMM Wolff K Balfour J Molecular analysis of plant migration and refugia in the arctic Science 2000 289 1343 1346 10.1126/science.289.5483.1343 10958779

[B51] Brochmann C Brysting AK Alsos IG Borgen L Grundt HH Scheen A-C Elven R Polyploidy in arctic plants Biol Jour Linn Soc 2004 82 521 536 10.1111/j.1095-8312.2004.00337.x

[B52] Brysting AK Mathiesen C Marcussen T Challenges in polyploid phylogenetic reconstruction: A case story from the arctic-alpine Cerastium alpinum complex TAXON 2011 60 333 347

[B53] Ehrendorfer F *Achillea roseoalba *Ehrendf., spec. nov., eine hybridogene, di- und tetraploide Sippe des *Achillea millefolium*-Komplexs Österr Bot Zeitschr 1959 106 363 368 10.1007/BF01289811

[B54] Vetter S Lambrou M Franz CH Ehrendorfer F Cytogenetics of experimental hybrids within the *Achillea millefolium *complex (yarrow) Caryologia 1996 49 1 12

[B55] Vetter S Lambrou M Franz CH Ehrendorfer F Saukel J Chromosome numbers of experimental tetraploid hybrids and selfpollinated progenies within the *Achillea millefolium *complex (Compositae) Caryologia 1996 49 227 231

[B56] Colinvaux PA The environment of the Bering Land Bridge Ecol Mono 1964 34 297 329 10.2307/1948504

[B57] Zuzula GD Froese DG Westgate JA La Farge C Mathewes RW Paleoecology of Beringian "packrat" middens from central Yukon Territory, Canada Q Res 2005 63 189 198 10.1016/j.yqres.2004.11.003

[B58] Guo Y-P Ehrendorfer F Samuel R Phylogeny and systematics of *Achillea *(Asteraceae-Anthemideae) inferred from nrITS and plastid *trnL-F *DNA sequences Taxon 2004 53 657 672 10.2307/4135441

[B59] Guo Y-P Vogl C Van Loo M Ehrendorfer F Hybrid origin and differentiation of two tetraploid *Achillea *species in East Asia: molecular, morphological and ecogeographical evidence Mol Ecol 2006 15 133 144 16367836 10.1111/j.1365-294X.2005.02772.x

[B60] Emerson KJ Merz CR Catchen JM Hohenlohe PA Cresko WA Bradshaw WE Holzapfel CM Resolving postglacial phylogeography using high-throughput sequencing Proc Natl Sci USA 2010 107 16196 16200 10.1073/pnas.1006538107 PMC2941283 20798348

[B61] Foll M Fischer MC Heckel G Excoffier L Estimating population structure from AFLP amplification intensity Mol Ecol 2010 19 4638 4647 10.1111/j.1365-294X.2010.04820.x 20874760

