# Supplementary material for: Nuclear and plastid haplotypes suggest rapid diploid and polyploid speciation in the N Hemisphere Achillea millefolium complex (Asteraceae)
Source: BMC Evol Biol. 2012 Jan 3;12:2. doi: 10.1186/1471-2148-12-2 (PMC3269993; doi:10.1186/1471-2148-12-2)
Supplement: Additional file 1 — The aligned polymorphic sites among 31 haplotypes of the sequenced ncpGS gene. These haplotypes are generated from 134 substitution sites among 186 clones (sequences) from 49 individuals of 20 populations of 10 diploid Achillea species (seven within and three outside A. millefolium agg.). Abbreviations of the species: (1) of A. millefolium agg.: asi = A. asiatica, asp = A. asplenifolia, cer = A. ceretanica, cus = A. cuspidate, lat = A. latiloba, ros = A. roseoalba and set = A. setacea; (2) of other species: acu = A. acuminata, lig = A. ligustica and nob = A. nobilis. Title of each haplotype sequence includes: abbreviation of species (number of populations/individuals/clones). Note: three haplotype of A. nobilis (nob) except the first one contain a recombination around the 89th polymorphic site between two or three haplotype groups of A. millefolium agg. (one above A. nobilis, and the others below). The sequences of A. cuspidate (cus) seems also containing a recombination around the 26th polymorphic site between those of A. ligustica (lig_SN) and some of A. millefolium agg. (e.g., several sequences at the top of the matrix). [file 1471-2148-12-2-S1.PDF]

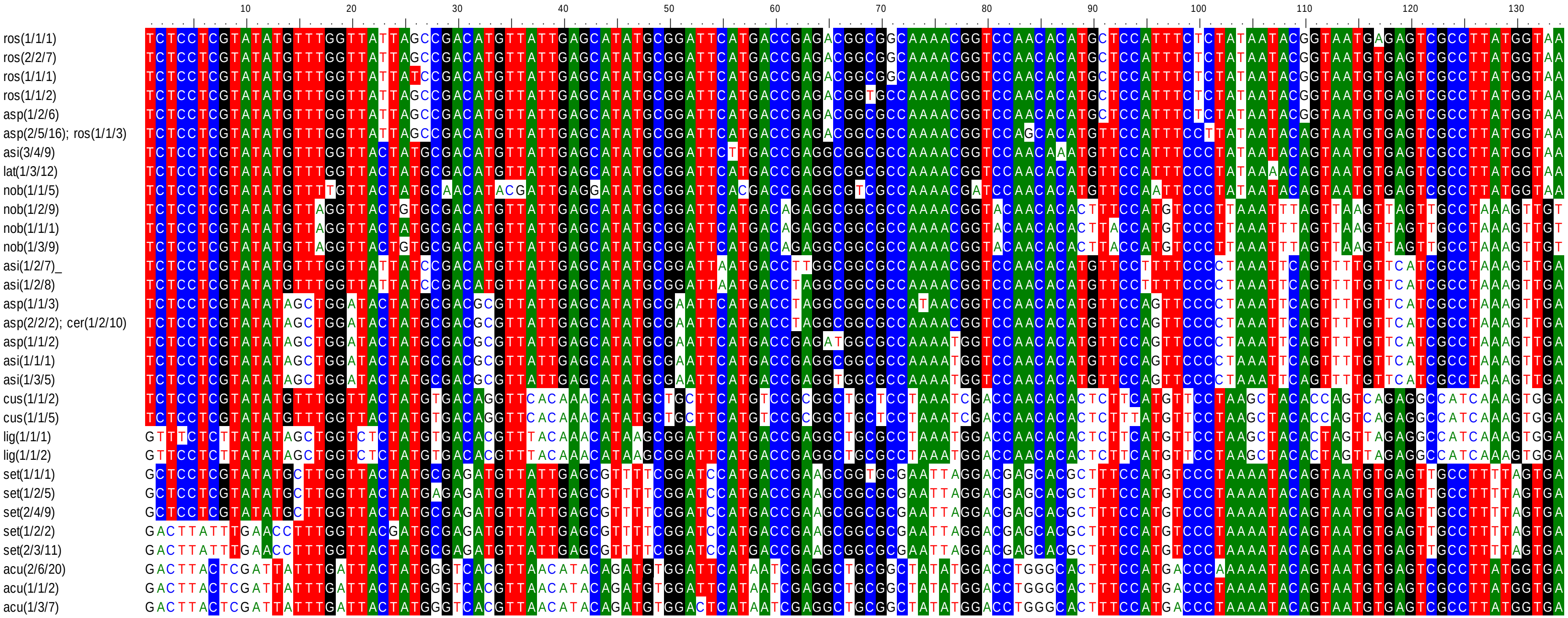

Additional file 1 S-Fig. 1 The aligned polymorphic sites among 31 haplotypes of the sequenced ncp*GS* gene. These haplotypes are generated from 134 substitution sites among 186 clones (sequences) from 49 individuals of 20 populations of 10 diploid *Achillea* species (seven within and three outside *A. millefolium* agg.). Abbreviations of the species: (1) of *A. millefolium* agg.: asi = *A. asiatica*, asp = *A. asplenifolia*, cer = *A. ceretanica*, cus = *A. cuspidate*, lat = *A. latiloba*, ros = *A. roseoalba* and set = *A. setacea*; (2) of other species: acu = *A. acuminata*, lig = *A. ligustica* and nob = *A. nobilis*. Title of each haplotype sequence includes: abbreviation of species (number of populations/individuals/clones).

Note: three haplotype of *A. nobilis* (nob) except the first one contain a recombination around the 89th polymorphic site between two or three haplotype groups of *A. millefolium* agg. (one above *A. nobilis*, and the others below). The sequences of *A. cuspidate* (cus) seems also containing a recombination around the 26th polymorphic site between those of *A. ligustica* (lig_SN) and some of *A. millefolium* agg. (e.g., several sequences at the top of the matrix).
