# Supplementary material for: Nuclear and plastid haplotypes suggest rapid diploid and polyploid speciation in the N Hemisphere Achillea millefolium complex (Asteraceae)
Source: BMC Evol Biol. 2012 Jan 3;12:2. doi: 10.1186/1471-2148-12-2 (PMC3269993; doi:10.1186/1471-2148-12-2)
Supplement: Additional file 2 — The aligned polymorphic sites among 21 SBP haplotypes. These haplotypes are generated from 60 substitution sites among 163 clones (sequences) from 35 individuals of 19 populations of 10 diploid Achillea species (seven within and three outside A. millefolium agg.). Abbreviations of the species: (1) of A. millefolium agg.: asi = A. asiatica, asp = A. asplenifolia, cer = A. ceretanica, cus = A. cuspidate, lat = A. latiloba, ros = A. roseoalba and set = A. setacea; (2) of other species: acu = A. acuminata, lig = A. ligustica and nob = A. nobilis. Title of each haplotype sequence includes: abbreviation of species (number of populations/individuals/clones). [file 1471-2148-12-2-S2.PDF]

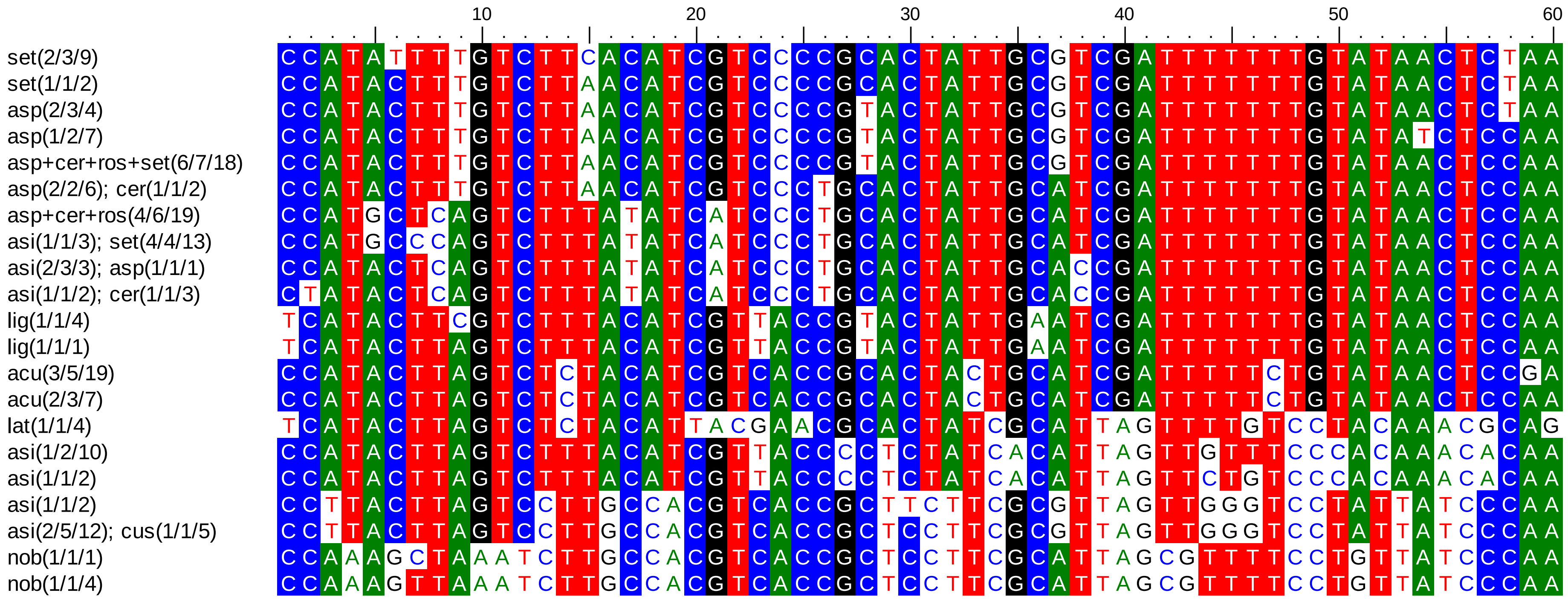

Additional file 2, S-Fig. 2 The aligned polymorphic sites among 21 *SBP* haplotypes. These haplotypes are generated from 60 substitution sites among 163 clones (sequences) from 35 individuals of 19 populations of 10 diploid *Achillea* species (seven within and three outside *A. millefolium* agg.). Abbreviations of the species: (1) of *A. millefolium* agg.: asi = *A. asiatica*, asp = *A. asplenifolia*, cer = *A. ceretanica*, cus = *A. cuspidate*, lat = *A. latiloba*, ros = *A. roseoalba* and set = *A. setacea*; (2) of other species: acu = *A. acuminata*, lig = *A. ligustica* and nob = *A. nobilis*. Title of each haplotype sequence includes: abbreviation of species (number of populations/individuals/clones).
