# Supplementary material for: Nuclear and plastid haplotypes suggest rapid diploid and polyploid speciation in the N Hemisphere Achillea millefolium complex (Asteraceae)
Source: BMC Evol Biol. 2012 Jan 3;12:2. doi: 10.1186/1471-2148-12-2 (PMC3269993; doi:10.1186/1471-2148-12-2)
Supplement: Additional file 3 — Unrooted Neighbour Joining cladogram of the SBP gene of all the diploid and polyploid taxa analyzed in this study. The tree contains 68 allelic haplotypes generated from 349 sequences with 68 substitution sites. Topology of the MP tree on the same data set is broadly comparable with that of the NJ tree. Bootstrap supports (> 50%) from NJ/MP analyses are shown next to the branches. Label of each terminal branch is written as "taxa abbreviation (population code-number of individuals/number of clones)". For taxa abbreviations, see Table 1. Diploid taxa are in black, polyploid taxa in different colors. [file 1471-2148-12-2-S3.PDF]

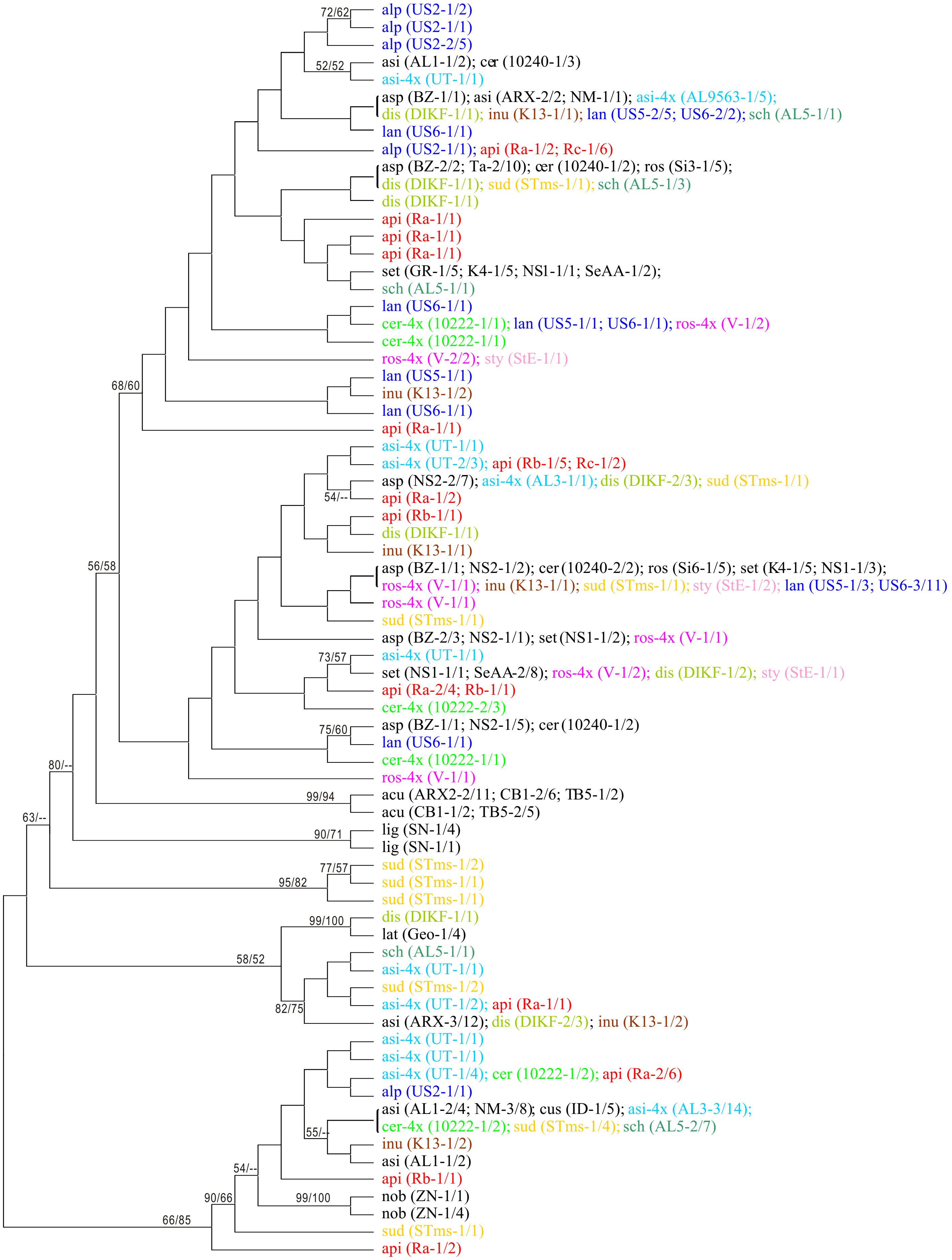

Additional file, 3 S-Fig. 3 Unrooted Neighbour Joining cladogram of the *SBP* gene of all the diploid and polyploid taxa analyzed in this study. The tree contains 68 allelic haplotypes generated from 349 sequences with 68 substitution sites. Topology of the MP tree on the same data set is broadly comparable with that of the NJ tree. Bootstrap supports (>50%) from NJ / MP analyses are shown next to the branches. Label of each terminal branch is written as "taxa abbreviation (population code-number of individuals / number of clones)". For taxa abbreviations, see Table 1. Diploid taxa are in black, polyploid taxa in different colors.
