# Supplementary figures and images for: Nuclear and plastid haplotypes suggest rapid diploid and polyploid speciation in the N Hemisphere Achillea millefolium complex (Asteraceae)
Source: BMC Evol Biol. 2012 Jan 3;12:2. doi: 10.1186/1471-2148-12-2 (PMC3269993; doi:10.1186/1471-2148-12-2)

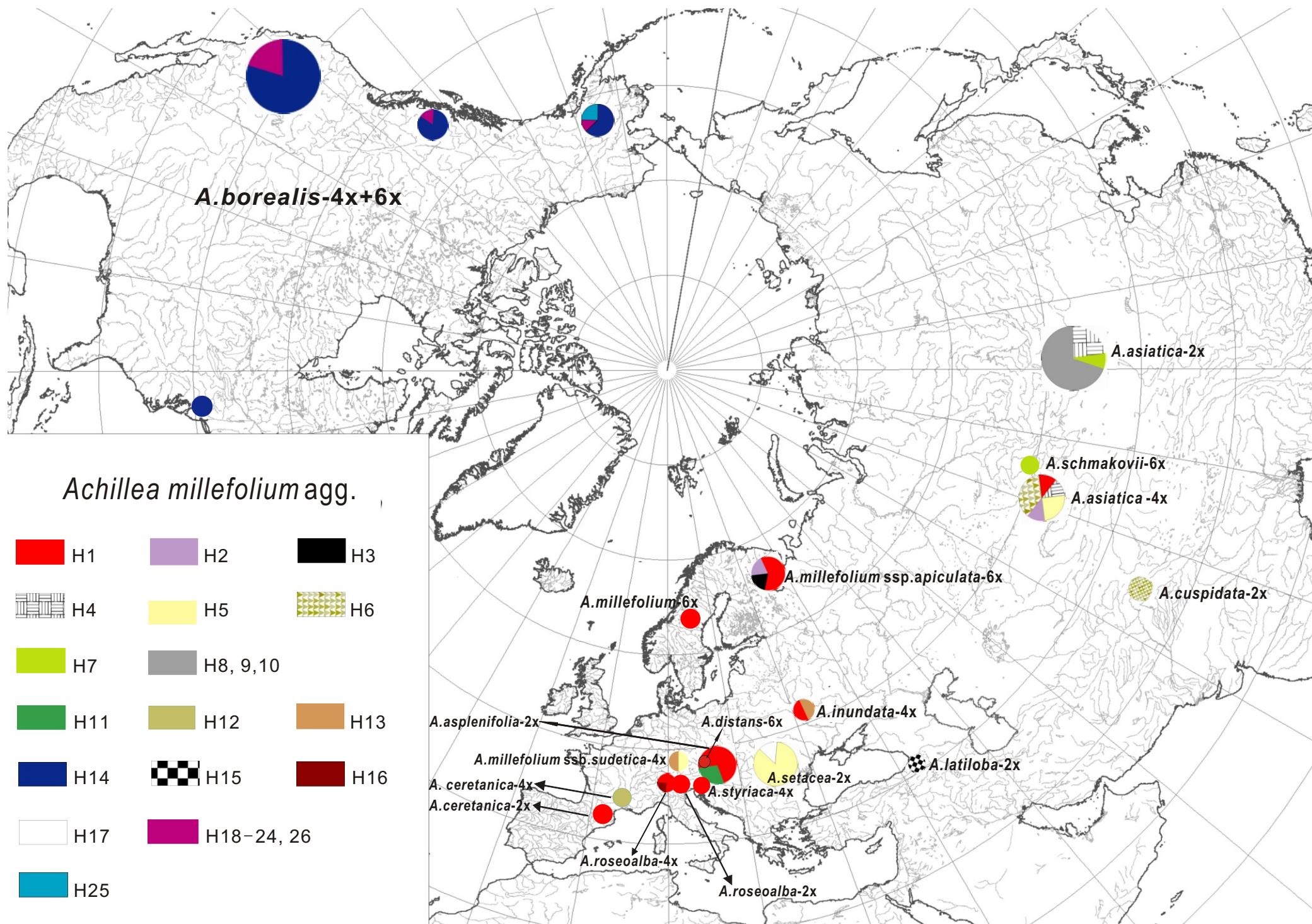

Supplement: Additional file 4 — Map showing the approximate distribution the 26 plastid haplotypes (H1-26) recognized across the temperate N Hemisphere. Pies indicate the proportion of haplotypes registered for individual taxa/cytotypes and the size of each pie correlated to the sample size from their generalized sampling areas. [file 1471-2148-12-2-S4.PDF]
